# Levels of Polonium-210 in brain and pulmonary tissues: Preliminary study in autopsies conducted in the city of Sao Paulo, Brazil

**DOI:** 10.1038/s41598-019-56973-z

**Published:** 2020-01-13

**Authors:** Nathalia Villa dos Santos, Carolina Leticia Zilli Vieira, Paulo Hilario Nascimento Saldiva, Barbara Paci Mazzilli, Mitiko Saiki, Catia Heloisa Saueia, Carmen Diva Saldiva De André, Lisie Tocci Justo, Marcelo Bessa Nisti, Petros Koutrakis

**Affiliations:** 1 Laboratory of Experimental Air Pollution, Department of Pathology, University of Sao Paulo School of Medicine, São Paulo, SP Brazil; 2 Department of Environmental Health, Harvard T.H. Chan School of Public Health, Boston, MA USA; 3 Nuclear and Energy Research Institute, IPEN-CNEN, São Paulo, SP Brazil; 4 Institute of Mathematics and Statistics, University of Sao Paulo, Sao Paulo, Brazil

**Keywords:** Neurological disorders, Risk factors

## Abstract

The accumulation of detectable amounts of radon progeny in human tissues may be a risk factor for development and progression of chronic diseases. In this preliminary study, we analyzed the levels of alpha-emitting radon progeny Polonium-210 (^210^Po) in the olfactory epithelium, olfactory bulb, frontal lobe, and lung tissues in cadavers from the city of Sao Paulo, SP, Brazil. We also assessed the association between ^210^Po levels and exposure parameters for urban air pollution using linear regression models adjusted for age, sex, smoke, time living in Sao Paulo, daily commuting, socioeconomic index, and anthracosis (traffic-related black carbon accumulation in the pleural region and in lymph). Our findings show that the concentration of ^210^Po was associated with anthracosis in lungs of non-smokers (coefficient = 6.0; standard error = 2.9; *p* = 0.04). Individuals with lower socioeconomic status also had significantly higher ^210^Po levels in lungs (coefficient = −1.19; standard error = 0.58; *p* = 0.042). The olfactory bulb had higher ^210^Po levels than either olfactory epithelium (*p* = 0.071), frontal lobe (*p* < 0.001), or lungs (*p* = 0.037). Our findings of the deposition of ^210^Po in autopsy tissues suggest that airborne radionuclides may contribute to the development of chronic diseases, including neurodegenerative diseases.

## Introduction

Sao Paulo is the largest and most populous city in Latin America and presents high levels of airborne particulate matter (PM), in which approximately 75% are contributed by vehicular traffic^1^. Many developing urban cities have four to fifteen times higher PM level averages than levels established by the World Health Organization (WHO) Air Quality Guidelines in 2016^2^. Exposures to PM with sizes ≤2.5 μm (PM_2.5_) are considered to cause about 3.8 million annual premature deaths worldwide, in which approximately 80% of those deaths are due heart disease and stroke, and 20% are from chronic respiratory diseases and other diseases including cancer (WHO, 2016)^3^. Air pollution has also been linked to increased risks for neuroinflammatory and neurodegenerative disease in different populations^4,5^.

A precise assessment of personal exposures to environmental risk factors is critical for the establishment of cause-effect relationship between exposure and disease, as a basis for developing public health policies^6,7^. A recent autopsy-based study suggested that the concentration of black carbon (BC) in pleural tissues could be used as a potential indicator of lifetime exposure to urban air pollution in the city of Sao Paulo, Brazil^6^. BC has been associated with decreased cognitive function in the elderly^8^. The accumulation of BC in the lungs and in lymph leads to an asymptomatic and milder type of pneumoconiosis called anthracosis^9^, which has also been used as an indicator of air pollution exposure in urban populations^6^.

Moreover, radon (Rn) progeny have been found in autopsy-based individuals with neurodegenerative diseases^10,11^. Momčilović *et al*. observed that Rn progeny bound selectively to cigarette smoke components strongly accumulates in the central nervous system (CNS)^10^. Rn and its progeny tend to accumulate in high-carbon tissues such as lipids and protein^10–12^, eventually emitting radioactive decay products that induce deleterious biological effects such as DNA injury, cell function impairment or apoptosis, and contributing to the development of chronic disease and cancer^10–19^.

The largest fraction of human exposures to environmental ionizing radiation is associated with the inhalation of Rn and its progeny^20^. Rn is a naturally occurring radioactive gas produced during the uranium (U) and thorium (Th) decay chain in the Earth’s crust. Rn is the most significant radioisotope in ^238^U decay-series due to its relatively long half-life, high mobility (as a Noble gas) and significant air concentration. Rn decay chain produces a series of short- and long-lived alpha and beta particle emitting progeny, including polonium-210 (^210^Po) and lead-210 (^210^Pb), respectively. Alpha particles are helium nuclei consisting of two protons and two neutrons with strong biological toxicity and penetrate only a short distance into tissues, while beta particles are electrons or positrons with less damage potential and deeper penetration^20^.

The air concentration of Rn and its progeny is affected by geological processes, meteorological conditions, and the number and size of atmospheric PM. These factors play a major role in the environmental transport and fate of airborne radioactivity^20^. Short- and long-lived radon progeny are rapidly attached to the surface of atmospheric PM^20^, reducing the fraction of the unattached radon and increasing the concentration of attached radon decay products to PM called PM radioactivity (PR)^20^. A study in São Paulo showed a large variation of alpha particle emissions from indoor radon and progeny with a mean of 147 Bq/m^3^ during the winter of 2003. WHO (2010) estimates a 10–20% increase in lung cancer risk per 100 Bq/m^3^ radon concentration^21^.

While higher concentrations of BC have been identified in lung autopsies in urban residents^6^, very little information about the levels of Rn progeny in different autopsy tissues is described in the literature. The quantification of Rn progeny in autopsies can be a useful method to improve scientific knowledge of chronic disease development and progression in urban areas, and help reveal new aspects of environmental risk factors. In this study, we measured the levels of ^210^Po in nasal epithelium, olfactory bulb, frontal lobe, and lung tissues in cadavers from Post Mortem Verification Service of the City of São Paulo (SVOC), São Paulo, Brazil. We compared the levels of ^210^Po between male and female, as well as smokers versus non-smokers. We also assessed the association between ^210^Po concentrations in tissues and exposure parameters for traffic-related air pollution.

## Results

**Figure 1:**
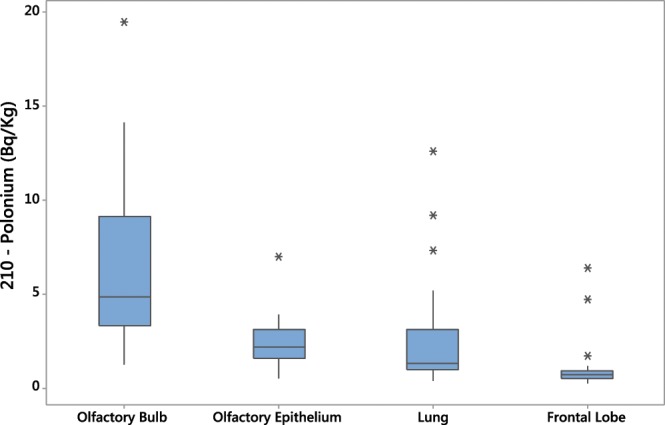
Polonium-210 concentrations in olfactory bulb, olfactory epithelium, lung and frontal lobe in the study population.

We studied 30 cadavers with a mean age of 66.0 (SD = 18.2) years, in which 40% (n = 12) were smokers and 56% (n = 17) were male. The average number of years living in Sao Paulo was 47.0 (SD = 16.1). A complete population description is presented in Table 1. Figure 1 shows the levels of ^210^Po (Bq/kg) in different tissues for all 30 cases. The levels of ^210^Po in the olfactory bulb were significantly higher than in the lung (*p* = 0.037), in the frontal lobe (*p* < 0.001) and also higher in the olfactory epithelium (*p* = 0.071).

**Table 1:** Descriptive statistics of 30 cases included in the study.

Variable	N (%)	Mean	SD	Minimum	Median	Maximum	IQR
Age (years)	30	66.5	18.23	25	68	93	26.5
Male	17 (57)	—	—	—	—	—	—
Smoker	12 (40)	—	—	—	—	—	—
Time living in São Paulo (years)	30	46.9	16.2	0.3	48	69	18.8
Daily commuting (hours)	30	2.1	2.7	0	0.7	10	3.3
Smoking pack-years	29	11.15	18.96	0	0	60	22.8
Anthracosis index	25	0.19	0.137	0.002	0.176	0.548	0.196
Socioeconomic index	27	−0.18	0.39	−0.79	−0.29	0.81	0.51

SD: Standard Deviation; y: years; pack-years: number of cigarettes smoked per day/20) X (years as a smoker).

**Figure 2:**
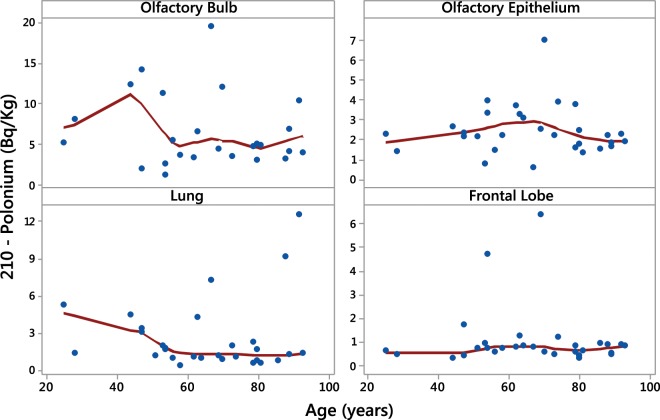
Polonium-210 concentrations in olfactory bulb, olfactory epithelium, lung and frontal lobe and age.

**Figure 3:**
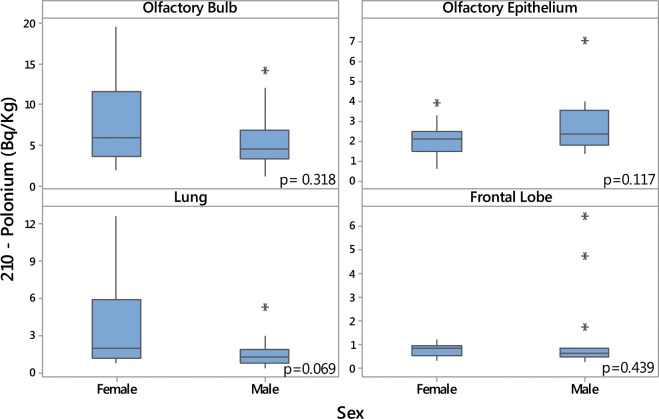
Polonium-210 concentrations in olfactory bulb, olfactory epithelium, lung and frontal lobe in females and males.

**Figure 4:**
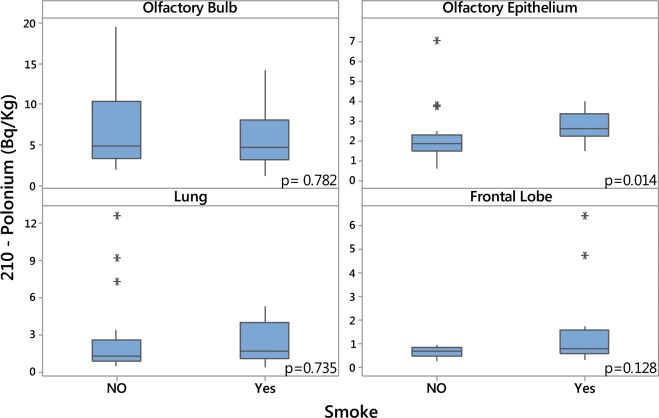
Polonium-210 concentrations in olfactory bulb, olfactory epithelium, lung and frontal lobe in smokers and non-smokers.

There was no statistically significant association between ^210^Po levels and age (Fig. 2). In comparison with males, females showed higher levels of ^210^Po in the olfactory bulb, lung and frontal lobe tissues, and lower levels in the olfactory epithelium (Fig. 3), but this difference was only statistically significant for the lungs (Fig. 3). Smokers presented higher concentrations of ^210^Po in the olfactory epithelium in comparison with non-smokers (*p* = 0.014) (Fig. 4). Smokers also had higher ^210^Po concentrations in the frontal lobe and the lungs in comparison with non-smokers; however, these differences were not statistically significant. There was no significant difference between ^210^Po levels in the olfactory bulb between smokers and non-smokers (Fig. 4).

Our regression modeling found a statistically significant association between ^210^Po levels and anthracosis index in lung tissues among non-smokers (coefficient = 6.0; standard error = 2.9; *p* = 0.040). The concentration of ^210^Po was also significantly higher in individuals with lower socioeconomic status (coefficient = −1.19; standard error = 0.58; *p* = 0.042). There was no significant association between the concentration of ^210^Po and time living in Sao Paulo and daily commuting (see details in the Supplementary Material).

## Discussion

This is the first study to investigate the concentration of radon progeny ^210^Po in cadaver tissues in a large metropolitan city (Sao Paulo, Brazil). Our results show that olfactory bulbs presented higher concentrations of ^210^Po in comparison to the other examined tissues. While women presented higher ^210^Po levels in the olfactory bulb, frontal lobe and lung tissues than men, there was no statistically significant difference in ^210^Po concentrations between genders. Smokers presented higher ^210^Po concentrations than non-smokers in the olfactory epithelium, frontal lobe and lungs, but this difference was only statistically significant for the olfactory epithelium.

The significant association between ^210^Po levels and anthracosis index in non-smoker’s lung tissues may suggest that the source of ^210^Po in these individuals was due to higher traffic-related urban pollution exposures. Takano *et al*. showed that pleural anthracosis deposition was higher in smokers in comparison with non-smokers in 413 autopsy-based cases^6^. Moreover, the concentration of ^210^Po was also higher in individuals with lower socioeconomic status. Previous studies showed that individuals with lower socioeconomic status are more exposed and vulnerable to hazardous air pollution in both urban and rural areas, including air particulates and radon^22–25^. Our findings of no significant association between ^210^Po levels and anthracosis among smokers’ lung tissues may be a consequence of the relatively small sample size.

Besides the environmental exposures to gaseous Rn and its particulate progeny^20^, higher concentrations of ^210^Po in smoker’s tissues found in our results may be due to ^210^Po in fertilizers used to grow tobacco plants^26^. Robert-Csaba Begy *et al*. estimated that approximately 63.41% of tobacco smoke ^210^Po reaches the respiratory system^27^, of which about 50% is retained in smoker’s lungs^28^. The average emission of ^210^Po in tobacco was 13.97 ± 1.75 mBq/non-filter cigarette in comparison with 1.61 ± 0.25 mBq/cigarette with filters, and 3.3 ± 0.29 mBq in the ash of a cigarette^27^. Increased ^210^Po deposition from tobacco smoke on bronchopulmonary tissues can induce carcinogenic activity in smokers and passive smokers, especially in individuals with compromised mucociliary clearance^26^.

Our finding that women had higher ^210^Po levels among different tissues than men are consistent with previous research. Ruano-Ravina *et al*. examined the association between residential radon levels and brain cancer mortality in Galicia, Spain, and showed that the risks were higher among women^29^. The authors attributed this to the fact that women spend more time in residential environments as compared to men^29^. Furthermore, the reduced capacity of pulmonary clearance observed in women and smoking individuals might explain our lung tissue findings. The absence of clearance mechanisms for the olfactory bulb can also explain the higher concentrations of ^210^Po in these tissues in comparison with lungs. Further CNS biochemical composition analyses could also add a substantial contribution to understanding the relatively high radon progeny affinity of some particular biological structures such as lipids and proteins^10–12^.

The transport of radon and its progeny from ambient air to the CNS may involve neuronal pathways via the olfactory system as demonstrated by previous studies for other air pollutants^30^. Oberdörster *et al*. showed that the olfactory bulb was the target for the deposition of inhaled ultrafine particles (diameter <100 nm), which are transported through the nasal region and olfactory nerve into the CNS^30^. Moreover, other studies have shown that both the olfactory nerve and the olfactory bulb are common pathways of heavy metal deposition into the CNS^26,31,32^. Our findings show higher concentrations of ^210^Po in olfactory bulbs, indicating that they may be on the major translocation pathway of radon progeny from the nasal tissues to the CNS.

Similar ^210^Po concentrations in the frontal lobe were previous reported in Momčilović *et al*. in a patient with Alzheimer’s disease^10^. The authors estimated that for 1,453 µBq/g approximately 160,000 cells are exposed to high-energy ionizing radiation from ^210^Po decay in the Hippocampus and the nucleus amygdala, potentially promoting the clinical progress towards dementia^10^. Our results show that the concentration of ^210^Po in the frontal lobe was 1,000 µBq/g and 6,400 µBq/g in the olfactory bulb. Dysfunction of the olfactory bulb has been associated with the development of neurodegenerative diseases such as Alzheimer’s and Parkinson’s^17^.

The increased permeability of olfactory epithelium with aging or inflammation leads to higher translocation of inhaled environmental agents from the olfactory nerve to the bulb, increasing the accumulation of markers of oxidative stress, inflammation, mitochondrial dysfunction and pathogenic proteins related to neurodegenerative disorders in CNS^17,18^. In addition, exposures to both low and high levels of ionizing radiation in the brain can directly change gene expression, synthesis and repair, inducing DNA breaks and apoptosis^18^. Brain cells are more susceptible to accumulation of unrepaired DNA lesions that induce neurocognitive dysfunctions that are characterized clinically by changes in learning processes and behavior, as well as chronic fatigue and depression^18^.

This preliminary study has limitations, including the relatively small number of subjects and the lack of quantification of personal exposures to environmental radon and air pollution. A study with a larger population could possibly show more significant associations between ^210^Po and personal characteristics, such as gender, age, and occupation. Nonetheless, our findings clearly show the presence of relatively high levels of environmental ^210^Po in different cadaver tissues in individuals who lived in a large metropolitan city. The accumulation of ^210^Po was higher in the olfactory bulb than in the olfactory epithelium, frontal lobe or lungs, which may be related to the lack of clearance mechanisms for the olfactory bulb and/or to its particular biochemical composition and high affinity to radon progeny as elucidated by previous studies^10–12^. Our findings of the deposition of ^210^Po in autopsy tissues suggest that airborne radionuclides may contribute to the development of chronic diseases, including neurodegenerative diseases.

## Methods

Fresh tissues of brain, lung, olfactory bulb and olfactory epithelium were obtained from 30 individuals from the Post-Mortem Verification Service of São Paulo city (SVOC) (Table 1). In an SVO routine, a relative [next-of-kin (NOK)] claimed the body. A trained interviewer invited the NOK to participate in the study. Upon acceptance, the relative was invited to a private room where an informed consent form was signed authorizing the collection of tissue samples (Supplementary Material). Subsequently, a questionnaire was used to determine previous health conditions, residential address, sociodemographic details, life habits, smoking habits, occupation, time of residence in the Metropolitan Sao Paulo area (MSP), and time spent commuting. The inclusion criteria were: age equal or greater than 18 years, living in the MSP area for at least 3 months, having one close relative to provide reliable and complete information during the interview, and the absence of macroscopic alterations of the lungs, brain or the olfactory epithelium. This study was approved by the Research Ethics Committee of the University of Sao Paulo (number 2013/21728).

### Sample preparation of human tissues

The tissue samples from brain (frontal lobe) and olfactory bulb, olfactory epithelium, lung (upper lobe) were placed separately in clean polyethylene bags and kept at −80 °C prior to transport to the Nuclear and Energy Research Institute (IPEN-CNEN/SP). Special care was taken during handling to avoid contamination using plastic tools and powder-free surgical gloves. The tissues were rinsed with purified water to remove the blood. Subsequently, the tissues were homogenized using a titanium knife and a hammer to grind cartilage of the olfactory epithelium. Samples were freeze-dried until their constant weight was obtained. In this process of freeze-drying mean percent weight losses were: 81.0 ± 7.1 for olfactory bulb, 71.9 ± 4.6 for olfactory epithelium, 82.0 ± 6.0 for lung and 79.7 ± 3.0 for brain. The dried samples were ground to a fine powder and placed in polyethylene vials, which were stored in a refrigerator.

### Determination of ^210^Po in human samples

There are a limited number of reliable methods available for the determination of ^210^Po, the most commonly used being alpha particle spectrometry, due to its sensitivity and selectivity^33^. For the determination of ^210^Po in the tissue samples, approximately 0.5 g of each sample (dry weight) was used. Prior to the analysis, 100 μL of ^209^Po tracer of known activity (1.648 Bq/g) was added. For the acid dissolution of the sample, 10 mL of concentrated HNO_3_ and hydrogen peroxide were added, under heating at 80 °C to avoid loss by volatilization of polonium isotopes. The solution was evaporated carefully to near dryness. This procedure was repeated until complete dissolution of the sample. The final solution was treated with concentrated HCl to eliminate nitrates. The final residue was dissolved in 0.5 M HCl and filtered with Millipore 0.1 μm membrane filters; 20% hydroxylamine hydrochloride was added to the solution. The pH was adjusted to 1.5 and the polonium was plated on a silver disc at 80 °C for 4 h, with agitation of the solution. The prepared sources were counted in a Canberra Alpha Analyst surface barrier detector for 150,000 s. For the determination of the detector alpha particle counting efficiency, a calibrated source of ^241^Am from Amersham, with activity of 5.55 kBq, was counted for 300 s. The counting efficiency of the detector varied from 22.54% to 26.96%.

### Urban pollution exposures

We analyzed the association between ^210^Po and the following potential predictors of exposure to urban pollution: time living in Sao Paulo, daily commuting, and anthracosis index^6^. Anthracosis index was measured *on* the pleural surface of the lungs. During the autopsy, lungs were removed and the excess of blood from the pleural surface was cleaned. Then a 10 cm Petri dish was superimposed on the anterior surfaces of the upper and lower lobes of both lungs to flatten the observation area. PhotoFigures of the pleura surface were taken with a high-resolution camera (Canon Power Shot SX400 IS). Using these images, the Fraction Area of Anthracosis (FA) was estimated by the point counting method^34^ with the aid of the software ImageJ (https://imagej.nih.gov/ij/docs/intro.html). For each of the four lobes, points falling on black patches (BP) and in the clean pleura (NBP) were counted separately and the fraction of anthracosis (FA) for each lung lobe determined applying the following formula:

$$FA=number\,of\,BP/(number\,of\,BP+number\,of\,NBP).$$

### Other parameters

We also assessed the association between ^210^Po and smoking habits (smoker versus non-smoker), and socioeconomic index. We included socioeconomic index^35^ to identify vulnerable conditions related to health in the city of Sao Paulo. This index is based on 27 variables related to poverty, wealth, education, income, social and material deprivation, and cultural aspects^35^. This index ranges from −1 to 1, with higher values indicating better socioeconomic conditions. Details of these assessment methods were described elsewhere^35^.

### Statistical analysis

All measurements of alpha particle activities from ^210^Po decay were expressed in Becquerel per kg of tissue (Bq/kg). Spearman’s correlation coefficient was used to evaluate the correlation between ^210^Po levels in the olfactory epithelium, olfactory bulb, frontal lobe, and lung tissues. Friedman’s test was applied to compare the distributions of ^210^Po in the different tissues. To find differences, pairwise comparisons were performed with Bonferroni correction. The Mann-Whitney test was applied to compare polonium distributions in male and female and in smokers and non-smokers. The association between the level of ^210^Po in the lung and anthracosis included adjustment for sex, age, smoking habits (smoker versus non-smoker), daily commuting (hr), years living in Sao Paulo, and socioeconomic index. Terms of interaction between sex and anthracosis and smoking and anthracosis were also added to the model. A residual analysis suggested the existence of one outlier. The analysis was repeated excluding this case. A robust regression model based on M-estimators was also considered. The model was fitted via the *rlm* function from the *MASS* library in the R software package. Due to the small sample size, the significance level was set at 0.10. The analysis was performed with Minitab (version 18) and R software package (R Development Core Team).

### Ethics statement

This study is part of the MetroHealth subproject of a project entitled The Use of Modern Autopsy Techniques to Investigate Human Diseases (MODAU) and was approved by the Research Ethics Committee of the University of Sao Paulo (number 2013/21728).

### Human experiment statement

All legal guardians signed the informed consent before the pathological procedures (Supplementary Material). This study was approved by the Research Ethics Committee of the University of Sao Paulo (number 2013/21728) in accordance with relevant regulations.

## Supplementary information

Supplementary Information

Final dataset.

## Data Availability

The datasets generated during and/or analyzed during the current study are available from the corresponding author on reasonable request.
