# Supplementary material for: Levels of Polonium-210 in brain and pulmonary tissues: Preliminary study in autopsies conducted in the city of Sao Paulo, Brazil
Source: Sci Rep. 2020 Jan 13;10:180. doi: 10.1038/s41598-019-56973-z (PMC6957520; doi:10.1038/s41598-019-56973-z)
Supplement: Supplementary file 1 — Supplementary Information [file 41598_2019_56973_MOESM1_ESM.docx]

**Supplementary Material**

**Paper: Levels of Polonium-210 in brain and pulmonary tissues: Preliminary study in autopsies conducted in the city of Sao Paulo. Brazil**

Nathalia Villa dos Santos^1§^, Carolina Leticia Zilli Vieira^2§*^, Paulo Hilario Nascimento Saldiva^1^, Barbara Paci Mazzilli^3^, Mitiko Saiki^3^, Catia Heloisa Saueia^3^, Carmen Diva Saldiva De André^4^, Lisie Tocci Justo^1^, Marcelo Bessa Nisti^3^, Petros Koutrakis^2^

^1^Laboratory of Experimental Air Pollution, Department of Pathology, University of Sao Paulo School of Medicine, São Paulo, SP, Brazil.

^2^Department of Environmental Health, Harvard T.H. Chan School of Public Health, Boston, MA. USA

^3^Nuclear and Energy Research Institute, IPEN-CNEN, São Paulo, SP, Brazil

^4^ Institute of Mathematics and Statistics, University of Sao Paulo, Sao Paulo, Brazil

**Supplementary Results**

**Table S1:** Concentration of ^210^Po in different tissues stratified by gender.

| **Tissue** | **^210^Po Levels (Bq/kg)** | | | | | |
| --- | --- | --- | --- | --- | --- | --- |
|  | **Male** | | **Female** | | **Total** | |
|  |  |  |  |  |  |  |
|  |  |  |  |  |  |  |
|  | **N** | **Mean (SD)** | **N** | **Mean (SD)** | **N** | **Mean (SD)** |
| **OB** | 16 | 5.76 (3.61) | 9 | 7.56 (5.72) | 25 | 6.41 (4.45) |
| **OE** | 18 | 2.71 (1.35) | 12 | 1.99 (0.95) | 30 | 2.42 (1.24) |
| **FL** | 18 | 1.23 (1.64) | 12 | 0.77 (0.29) | 30 | 1.04 (1.29) |
| **L** | 18 | 2.16 (2.84) | 12 | 3.09 (2.76) | 30 | 2.53 (2.80) |

**Table legend.** * ^210^Po levels expressed in Bq/kg; OB: Olfactory bulb; OE: Olfactory epithelium; FL: Frontal lobe; L: lungs; SD: Standard deviation

**Table S2:** Concentration of ^210^Po in different tissues stratified by age categories.

| **Age (years)** | **^210^Po Levels (Bq/Kg)** | | | | | | | |
| --- | --- | --- | --- | --- | --- | --- | --- | --- |
|  |  |  |  |  |  |  |  |  |
|  | **OB** | | **OE** | | **FL** | | **L** | |
|  | **N** | **Mean (SD)** | **N** | **Mean (SD)** | **N** | **Mean (SD)** | **N** | **Mean (SD)** |
|  |  |  |  |  |  |  |  |  |
| **25-39** | 2 | 6.54 (2.07) | 2 | 1.80 (0.61) | 2 | 0.53 (0.12) | 2 | 3.28 (2.76) |
| **40-54** | 6 | 7.26 (5.95) | 7 | 2.46 (1.02) | 7 | 1.36 (1.56) | 7 | 2.47 (1.17) |
| **55-69** | 6 | 7.11 (6.18) | 7 | 2.39 (1.10) | 7 | 1.61 (2.13) | 7 | 2.30 (2.55) |
| **70-84** | 6 | 5.47 (3.25) | 8 | 2.98 (1.89) | 8 | 0.62 (0.28) | 8 | 1.20 (0.67) |
| **>85** | 5 | 5.64 (2.96) | 6 | 1.88 (0.30) | 6 | 0.75 (0.21) | 6 | 4.41 (5.15) |
| **Total** | 25 | 6.41 (4.45) | 30 | 2.42 (1.24) | 30 | 1.04 (1.29) | 30 | 2.53 (2.80) |

**Table legend.** * ^210^Po levels expressed in Bq/kg; OB: olfactory bulb; OE: olfactory epithelium; FL: frontal lobe; L: lungs; SD: Standard Deviation

**Table S3:** Concentration of ^210^Po in different tissues stratified by smoking status.

| **Tissue** | **Po210 Levels (Bq/Kg)** | | | | | |
| --- | --- | --- | --- | --- | --- | --- |
|  | **No-Smoker** | | **Smoker** | | **Total** | |
|  |  |  |  |  |  |  |
|  | **N** | **Mean(SD)** | **N** | **Mean(SD)** | **N** | **Mean(SD)** |
|  |  |  |  |  |  |  |
| **OB** | 13 | 6.66 (4.96) | 12 | 6.13 (4.03) | 25 | 6.41 (4.45) |
| **OE** | 16 | 2.10 (1.49) | 14 | 2.78 (0.75) | 30 | 2.42 (1.23) |
| **FL** | 16 | 0.63 (0.20) | 14 | 1.51 (1.79) | 30 | 1.04 (1.28) |
| **L** | 16 | 2.14 (0.49) | 14 | 2.97 (3.14) | 30 | 2.53 (2.8) |

**Table legend.** * ^210^Po levels expressed in Bq/kg; OB: Olfactory bulb; OE: Olfactory epithelium; FL: Frontal lobe; L: lungs; SD: Standard deviation

**Table S4.** Spearman correlation coefficient among different tissues

|  | **Olfactory Bulb** | **Olfactory Epithelium** | **Lung** | **Frontal Lobe** |
| --- | --- | --- | --- | --- |
| **Olfactory Bulb** | 1 | -0.18 | 0.25 | 0.05 |
|  | . | 0.387 | 0.224 | 0.829 |
|  | 25 | 25 | 25 | 25 |
| **Olfactory Epithelium** | -0.18 | 1 | 0.13 | 0.30 |
|  | 0.387 | . | 0.49 | 0.108 |
|  | 25 | 30 | 30 | 30 |
| **Lung** | 0.25 | 0.13 | 1 | 0.14 |
|  | 0.224 | 0.490 | . | 0.461 |
|  | 25 | 30 | 30 | 30 |
| **Frontal Lobe** | 0.05 | 0.30 | 0.14 | 1 |
|  | 0.829 | 0.108 | 0.461 | . |
|  | 25 | 30 | 30 | 30 |

**Table legend.** ^210^Po levels expressed in Bq/kg.

**Table S5.** Spearman correlation coefficient between Age. Years living in Sao Paulo. Daily commuting and Socioeconomic index. Street density and Distance with 210-Polonium concentrations in Olfactory bulb. Olfactory Epithelium. Lung and Frontal Lobe

|  |  | Olfactory Bulb | Olfactory Epithelium | Lung | Frontal Lobe |
| --- | --- | --- | --- | --- | --- |
| Age | Correlation Coefficient | -0.137 | -0.138 | -0.193 | 0.024 |
|  | Sig. (2-tailed) | 0.514 | 0.467 | 0.306 | 0.899 |
|  | N | 25 | 30 | 30 | 30 |
| Time living in the city | Correlation Coefficient | **-0.458** | 0.073 | -0.297 | 0.053 |
|  | Sig. (2-tailed) | **0.021** | 0.703 | 0.111 | 0.782 |
|  | N | **25** | 30 | 30 | 30 |
| Commuting hours | Correlation Coefficient | -0.134 | 0.093 | -0.246 | 0.051 |
|  | Sig. (2-tailed) | 0.524 | 0.627 | 0.19 | 0.788 |
|  | N | 25 | 30 | 30 | 30 |
| Anthracosis | Correlation Coefficient | -0.298 | -0.095 | -0.058 | -0.116 |
|  | Sig. (2-tailed) | 0.202 | 0.65 | 0.784 | 0.58 |
|  | N | 20 | 25 | 25 | 25 |
| Socioeconomic index | Correlation Coefficient | -0.072 | **-0.346** | **-0.412** | -0.096 |
|  | Sig. (2-tailed) | 0.751 | **0.077** | **0.033** | 0.632 |
|  | N | 22 | 27 | 27 | 27 |

**Table legend.** ^210^Po levels expressed in Bq/kg.
